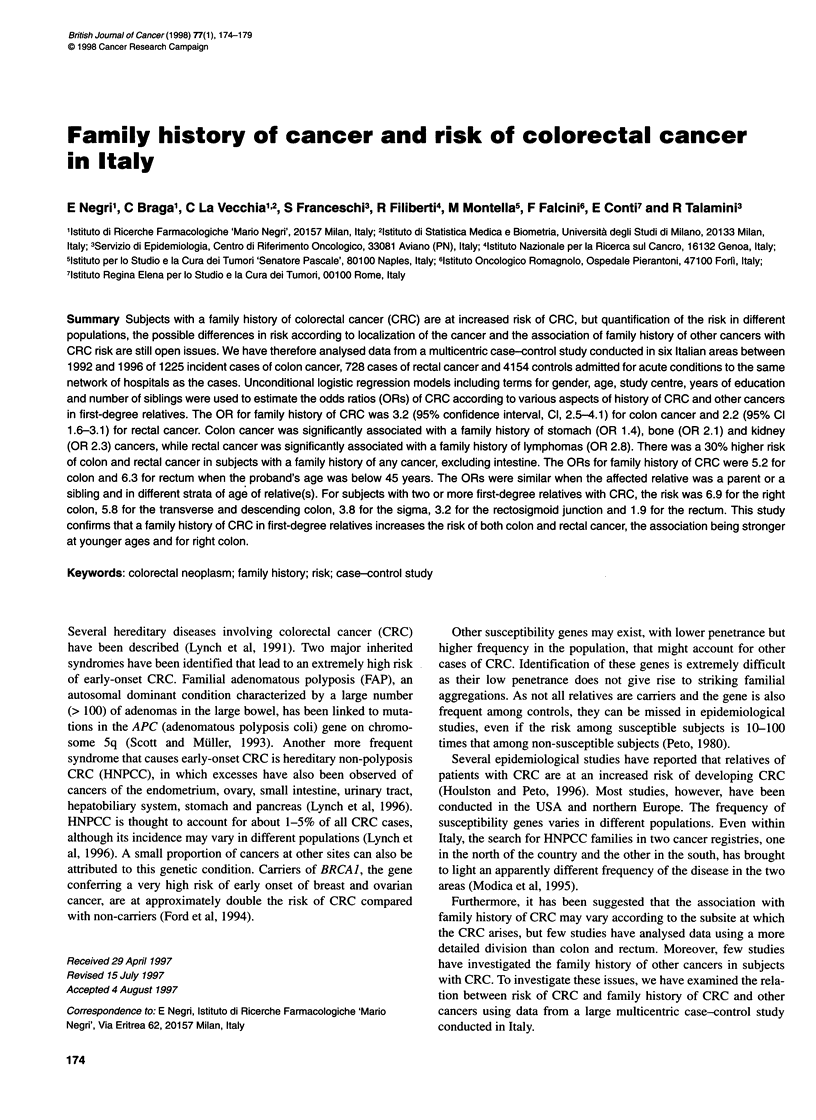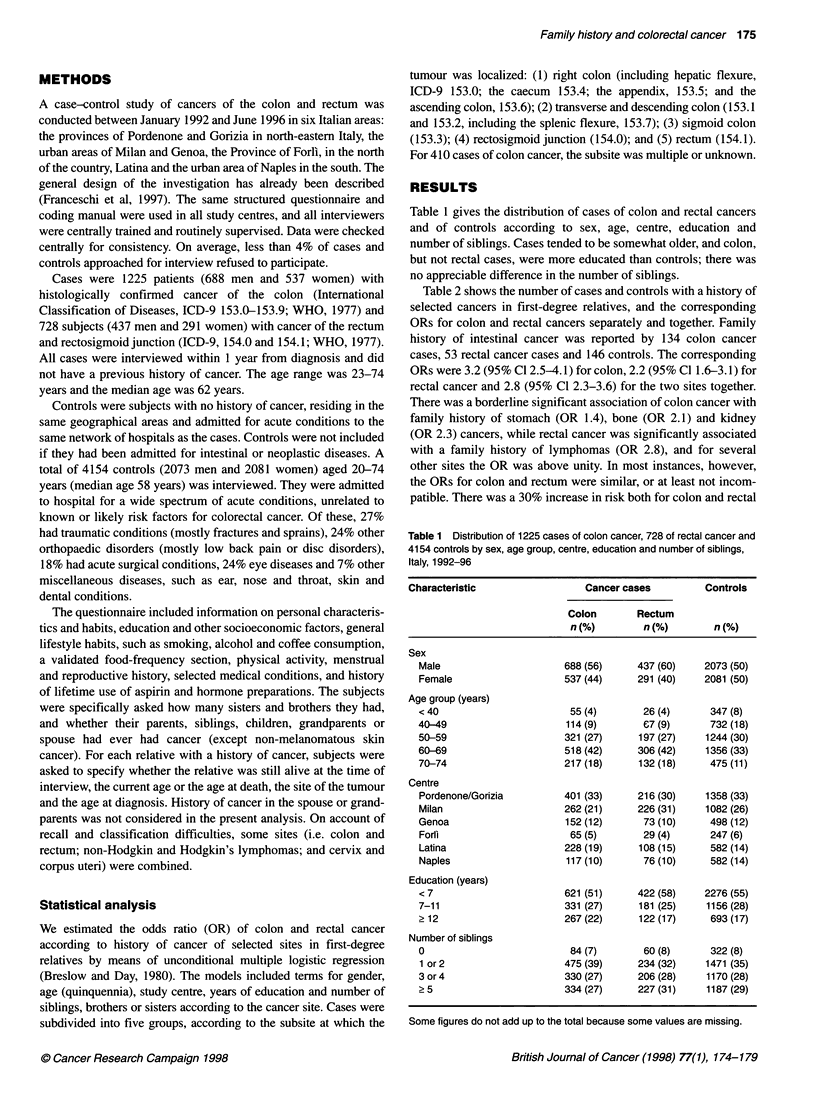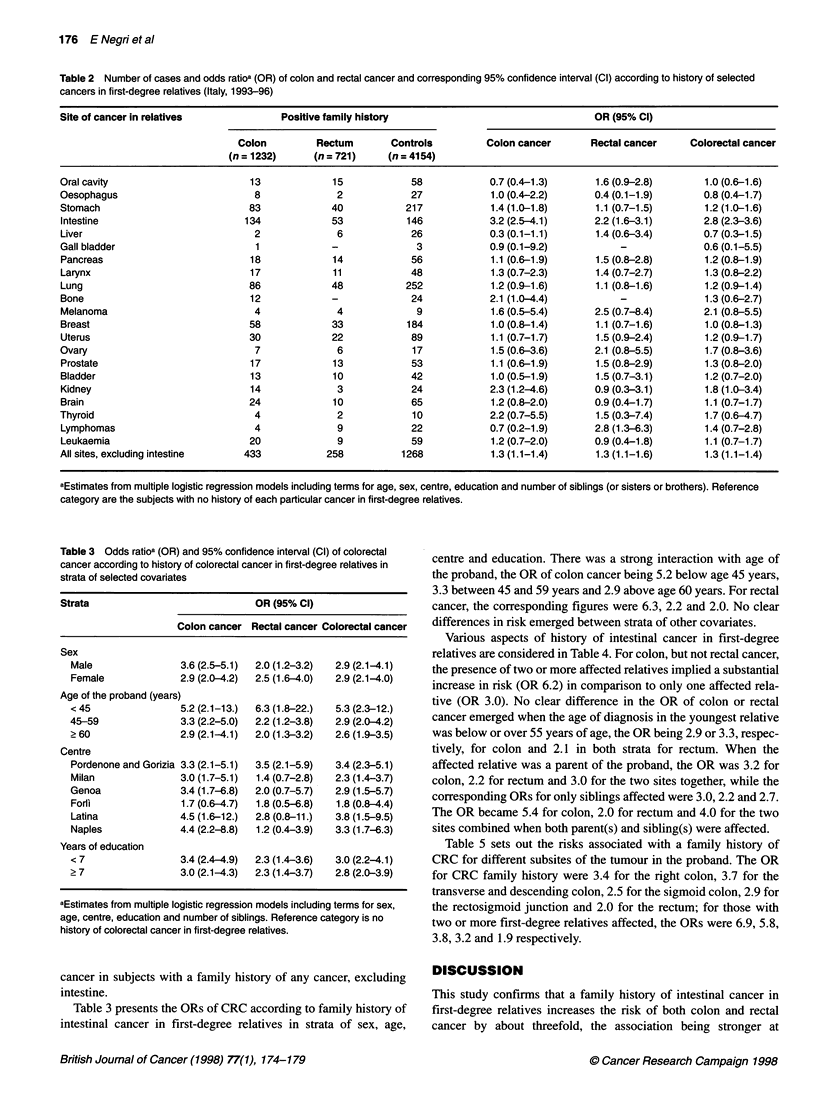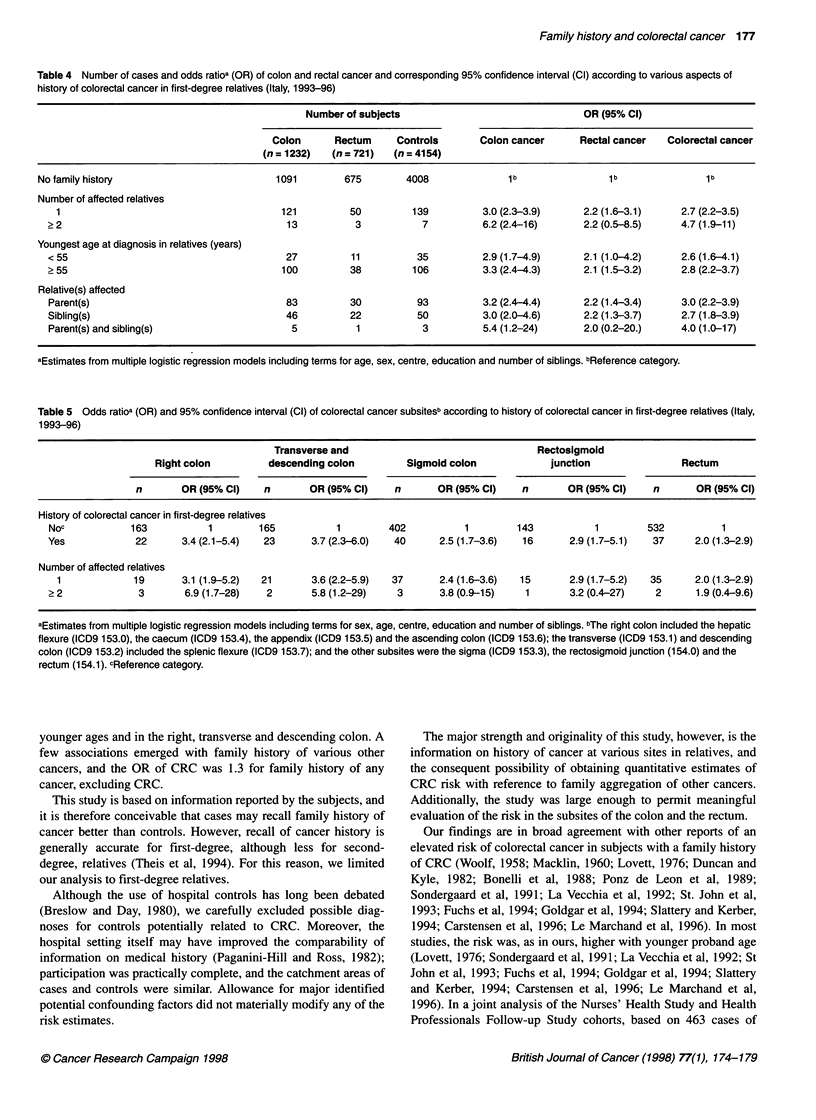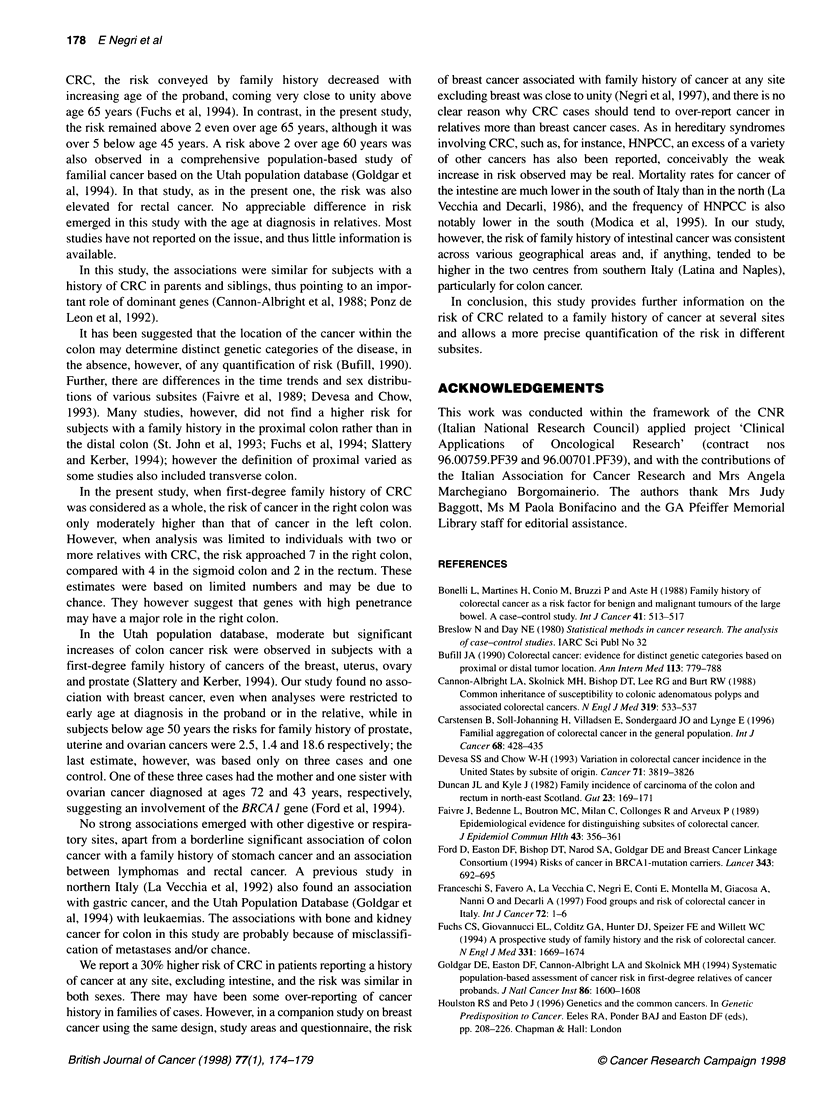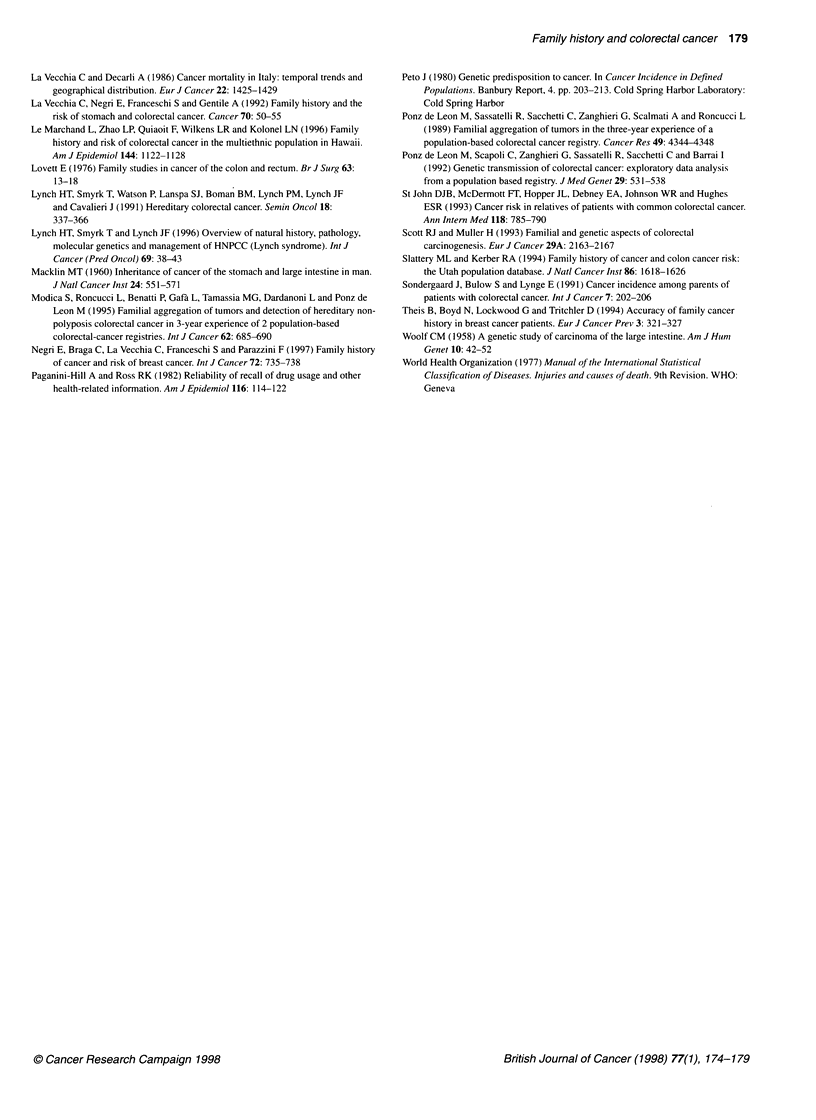# Family history of cancer and risk of colorectal cancer in Italy.

**DOI:** 10.1038/bjc.1998.28

**Published:** 1998

**Authors:** E. Negri, C. Braga, C. La Vecchia, S. Franceschi, R. Filiberti, M. Montella, F. Falcini, E. Conti, R. Talamini

**Affiliations:** Istituto di Ricerche Farmacologiche Mario Negri, Milan, Italy.

## Abstract

Subjects with a family history of colorectal cancer (CRC) are at increased risk of CRC, but quantification of the risk in different populations, the possible differences in risk according to localization of the cancer and the association of family history of other cancers with CRC risk are still open issues. We have therefore analysed data from a multicentric case-control study conducted in six Italian areas between 1992 and 1996 of 1225 incident cases of colon cancer, 728 cases of rectal cancer and 4154 controls admitted for acute conditions to the same network of hospitals as the cases. Unconditional logistic regression models including terms for gender, age, study centre, years of education and number of siblings were used to estimate the odds ratios (ORs) of CRC according to various aspects of history of CRC and other cancers in first-degree relatives. The OR for family history of CRC was 3.2 (95% confidence interval, CI, 2.5-4.1) for colon cancer and 2.2 (95% CI 1.6-3.1) for rectal cancer. Colon cancer was significantly associated with a family history of stomach (OR 1.4), bone (OR 2.1) and kidney (OR 2.3) cancers, while rectal cancer was significantly associated with a family history of lymphomas (OR 2.8). There was a 30% higher risk of colon and rectal cancer in subjects with a family history of any cancer, excluding intestine. The ORs for family history of CRC were 5.2 for colon and 6.3 for rectum when the proband's age was below 45 years. The ORs were similar when the affected relative was a parent or a sibling and in different strata of age of relative(s). For subjects with two or more first-degree relatives with CRC, the risk was 6.9 for the right colon, 5.8 for the transverse and descending colon, 3.8 for the sigma, 3.2 for the rectosigmoid junction and 1.9 for the rectum. This study confirms that a family history of CRC in first-degree relatives increases the risk of both colon and rectal cancer, the association being stronger at younger ages and for right colon.


British Journal of Cancer (1998) 77(1), 174–179
© 1998 Cancer Research Campaign

### Family history of cancer and risk of colorectal cancer in Italy

**E Negri[1], C Braga[1], C La Vecchia[1,2], S Franceschi[3], R Filiberti[4], M Montella[5], F Falcini[6], E Conti[7] and R Talamini[3]**

[1]Istituto di Ricerche Farmacologiche 'Mario Negri', 20157 Milan, Italy; [2]Istituto di Statistica Medica e Biometria, Università degli Studi di Milano, 20133 Milan, Italy; [3]Servizio di Epidemiologia, Centro di Riferimento Oncologico, 33081 Aviano (PN), Italy; [4]Istituto Nazionale per la Ricerca sul Cancro, 16132 Genoa, Italy; [5]Istituto per lo Studio e la Cura dei Tumori 'Senatore Pascale', 80100 Naples, Italy; [6]Istituto Oncologico Romagnolo, Ospedale Pierantoni, 47100 Forlì, Italy; [7]Istituto Regina Elena per lo Studio e la Cura dei Tumori, 00100 Rome, Italy

**Summary** Subjects with a family history of colorectal cancer (CRC) are at increased risk of CRC, but quantification of the risk in different populations, the possible differences in risk according to localization of the cancer and the association of family history of other cancers with CRC risk are still open issues. We have therefore analysed data from a multicentric case–control study conducted in six Italian areas between 1992 and 1996 of 1225 incident cases of colon cancer, 728 cases of rectal cancer and 4154 controls admitted for acute conditions to the same network of hospitals as the cases. Unconditional logistic regression models including terms for gender, age, study centre, years of education and number of siblings were used to estimate the odds ratios (ORs) of CRC according to various aspects of history of CRC and other cancers in first-degree relatives. The OR for family history of CRC was 3.2 (95% confidence interval, CI, 2.5–4.1) for colon cancer and 2.2 (95% CI 1.6–3.1) for rectal cancer. Colon cancer was significantly associated with a family history of stomach (OR 1.4), bone (OR 2.1) and kidney (OR 2.3) cancers, while rectal cancer was significantly associated with a family history of lymphomas (OR 2.8). There was a 30% higher risk of colon and rectal cancer in subjects with a family history of any cancer, excluding intestine. The ORs for family history of CRC were 5.2 for colon and 6.3 for rectum when the proband's age was below 45 years. The ORs were similar when the affected relative was a parent or a sibling and in different strata of age of relative(s). For subjects with two or more first-degree relatives with CRC, the risk was 6.9 for the right colon, 5.8 for the transverse and descending colon, 3.8 for the sigma, 3.2 for the rectosigmoid junction and 1.9 for the rectum. This study confirms that a family history of CRC in first-degree relatives increases the risk of both colon and rectal cancer, the association being stronger at younger ages and for right colon.

**Keywords**: colorectal neoplasm; family history; risk; case–control study

Several hereditary diseases involving colorectal cancer (CRC) have been described (Lynch et al, 1991). Two major inherited syndromes have been identified that lead to an extremely high risk of early-onset CRC. Familial adenomatous polyposis (FAP), an autosomal dominant condition characterized by a large number (> 100) of adenomas in the large bowel, has been linked to mutations in the *APC* (adenomatous polyposis coli) gene on chromosome 5q (Scott and Müller, 1993). Another more frequent syndrome that causes early-onset CRC is hereditary non-polyposis CRC (HNPCC), in which excesses have also been observed of cancers of the endometrium, ovary, small intestine, urinary tract, hepatobiliary system, stomach and pancreas (Lynch et al, 1996). HNPCC is thought to account for about 1–5% of all CRC cases, although its incidence may vary in different populations (Lynch et al, 1996). A small proportion of cancers at other sites can also be attributed to this genetic condition. Carriers of *BRCA1*, the gene conferring a very high risk of early onset of breast and ovarian cancer, are at approximately double the risk of CRC compared with non-carriers (Ford et al, 1994).

*Received 29 April 1997*
*Revised 15 July 1997*
*Accepted 4 August 1997*

*Correspondence to:* E Negri, Istituto di Ricerche Farmacologiche 'Mario Negri', Via Eritrea 62, 20157 Milan, Italy

Other susceptibility genes may exist, with lower penetrance but higher frequency in the population, that might account for other cases of CRC. Identification of these genes is extremely difficult as their low penetrance does not give rise to striking familial aggregations. As not all relatives are carriers and the gene is also frequent among controls, they can be missed in epidemiological studies, even if the risk among susceptible subjects is 10–100 times that among non-susceptible subjects (Peto, 1980).

Several epidemiological studies have reported that relatives of patients with CRC are at an increased risk of developing CRC (Houlston and Peto, 1996). Most studies, however, have been conducted in the USA and northern Europe. The frequency of susceptibility genes varies in different populations. Even within Italy, the search for HNPCC families in two cancer registries, one in the north of the country and the other in the south, has brought to light an apparently different frequency of the disease in the two areas (Modica et al, 1995).

Furthermore, it has been suggested that the association with family history of CRC may vary according to the subsite at which the CRC arises, but few studies have analysed data using a more detailed division than colon and rectum. Moreover, few studies have investigated the family history of other cancers in subjects with CRC. To investigate these issues, we have examined the relation between risk of CRC and family history of CRC and other cancers using data from a large multicentric case–control study conducted in Italy.

### METHODS

A case–control study of cancers of the colon and rectum was conducted between January 1992 and June 1996 in six Italian areas: the provinces of Pordenone and Gorizia in north-eastern Italy, the urban areas of Milan and Genoa, the Province of Forlì, in the north of the country, Latina and the urban area of Naples in the south. The general design of the investigation has already been described (Franceschi et al, 1997). The same structured questionnaire and coding manual were used in all study centres, and all interviewers were centrally trained and routinely supervised. Data were checked centrally for consistency. On average, less than 4% of cases and controls approached for interview refused to participate.

Cases were 1225 patients (688 men and 537 women) with histologically confirmed cancer of the colon (International Classification of Diseases, ICD-9 153.0–153.9; WHO, 1977) and 728 subjects (437 men and 291 women) with cancer of the rectum and rectosigmoid junction (ICD-9, 154.0 and 154.1; WHO, 1977). All cases were interviewed within 1 year from diagnosis and did not have a previous history of cancer. The age range was 23–74 years and the median age was 62 years.

Controls were subjects with no history of cancer, residing in the same geographical areas and admitted for acute conditions to the same network of hospitals as the cases. Controls were not included if they had been admitted for intestinal or neoplastic diseases. A total of 4154 controls (2073 men and 2081 women) aged 20–74 years (median age 58 years) was interviewed. They were admitted to hospital for a wide spectrum of acute conditions, unrelated to known or likely risk factors for colorectal cancer. Of these, 27% had traumatic conditions (mostly fractures and sprains), 24% other orthopaedic disorders (mostly low back pain or disc disorders), 18% had acute surgical conditions, 24% eye diseases and 7% other miscellaneous diseases, such as ear, nose and throat, skin and dental conditions.

The questionnaire included information on personal characteristics and habits, education and other socioeconomic factors, general lifestyle habits, such as smoking, alcohol and coffee consumption, a validated food-frequency section, physical activity, menstrual and reproductive history, selected medical conditions, and history of lifetime use of aspirin and hormone preparations. The subjects were specifically asked how many sisters and brothers they had, and whether their parents, siblings, children, grandparents or spouse had ever had cancer (except non-melanomatous skin cancer). For each relative with a history of cancer, subjects were asked to specify whether the relative was still alive at the time of interview, the current age or the age at death, the site of the tumour and the age at diagnosis. History of cancer in the spouse or grandparents was not considered in the present analysis. On account of recall and classification difficulties, some sites (i.e. colon and rectum; non-Hodgkin and Hodgkin's lymphomas; and cervix and corpus uteri) were combined.

### Statistical analysis

We estimated the odds ratio (OR) of colon and rectal cancer according to history of cancer of selected sites in first-degree relatives by means of unconditional multiple logistic regression (Breslow and Day, 1980). The models included terms for gender, age (quinquennia), study centre, years of education and number of siblings, brothers or sisters according to the cancer site. Cases were subdivided into five groups, according to the subsite at which the tumour was localized: (1) right colon (including hepatic flexure, ICD-9 153.0; the caecum 153.4; the appendix, 153.5; and the ascending colon, 153.6); (2) transverse and descending colon (153.1 and 153.2, including the splenic flexure, 153.7); (3) sigmoid colon (153.3); (4) rectosigmoid junction (154.0); and (5) rectum (154.1). For 410 cases of colon cancer, the subsite was multiple or unknown.

### RESULTS

Table 1 gives the distribution of cases of colon and rectal cancers and of controls according to sex, age, centre, education and number of siblings. Cases tended to be somewhat older, and colon, but not rectal cases, were more educated than controls; there was no appreciable difference in the number of siblings.

Table 2 shows the number of cases and controls with a history of selected cancers in first-degree relatives, and the corresponding ORs for colon and rectal cancers separately and together. Family history of intestinal cancer was reported by 134 colon cancer cases, 53 rectal cancer cases and 146 controls. The corresponding ORs were 3.2 (95% Cl 2.5–4.1) for colon, 2.2 (95% Cl 1.6–3.1) for rectal cancer and 2.8 (95% Cl 2.3–3.6) for the two sites together. There was a borderline significant association of colon cancer with family history of stomach (OR 1.4), bone (OR 2.1) and kidney (OR 2.3) cancers, while rectal cancer was significantly associated with a family history of lymphomas (OR 2.8), and for several other sites the OR was above unity. In most instances, however, the ORs for colon and rectum were similar, or at least not incompatible. There was a 30% increase in risk both for colon and rectal

**Table 1** Distribution of 1225 cases of colon cancer, 728 of rectal cancer and 4154 controls by sex, age group, centre, education and number of siblings, Italy, 1992–96

| Characteristic | Cancer cases | | Controls |
|---|---|---|---|
| | Colon *n* (%) | Rectum *n* (%) | *n* (%) |
| Sex | | | |
| Male | 688 (56) | 437 (60) | 2073 (50) |
| Female | 537 (44) | 291 (40) | 2081 (50) |
| Age group (years) | | | |
| < 40 | 55 (4) | 26 (4) | 347 (8) |
| 40–49 | 114 (9) | 67 (9) | 732 (18) |
| 50–59 | 321 (27) | 197 (27) | 1244 (30) |
| 60–69 | 518 (42) | 306 (42) | 1356 (33) |
| 70–74 | 217 (18) | 132 (18) | 475 (11) |
| Centre | | | |
| Pordenone/Gorizia | 401 (33) | 216 (30) | 1358 (33) |
| Milan | 262 (21) | 226 (31) | 1082 (26) |
| Genoa | 152 (12) | 73 (10) | 498 (12) |
| Forlì | 65 (5) | 29 (4) | 247 (6) |
| Latina | 228 (19) | 108 (15) | 582 (14) |
| Naples | 117 (10) | 76 (10) | 582 (14) |
| Education (years) | | | |
| < 7 | 621 (51) | 422 (58) | 2276 (55) |
| 7–11 | 331 (27) | 181 (25) | 1156 (28) |
| ≥ 12 | 267 (22) | 122 (17) | 693 (17) |
| Number of siblings | | | |
| 0 | 84 (7) | 60 (8) | 322 (8) |
| 1 or 2 | 475 (39) | 234 (32) | 1471 (35) |
| 3 or 4 | 330 (27) | 206 (28) | 1170 (28) |
| ≥ 5 | 334 (27) | 227 (31) | 1187 (29) |

Some figures do not add up to the total because some values are missing.

© Cancer Research Campaign 1998

**Table 2** Number of cases and odds ratio[a] (OR) of colon and rectal cancer and corresponding 95% confidence interval (CI) according to history of selected cancers in first-degree relatives (Italy, 1993–96)

| Site of cancer in relatives | Positive family history | | | OR (95% CI) | | |
|---|---|---|---|---|---|---|
| | Colon (n = 1232) | Rectum (n = 721) | Controls (n = 4154) | Colon cancer | Rectal cancer | Colorectal cancer |
| Oral cavity | 13 | 15 | 58 | 0.7 (0.4–1.3) | 1.6 (0.9–2.8) | 1.0 (0.6–1.6) |
| Oesophagus | 8 | 2 | 27 | 1.0 (0.4–2.2) | 0.4 (0.1–1.9) | 0.8 (0.4–1.7) |
| Stomach | 83 | 40 | 217 | 1.4 (1.0–1.8) | 1.1 (0.7–1.5) | 1.2 (1.0–1.6) |
| Intestine | 134 | 53 | 146 | 3.2 (2.5–4.1) | 2.2 (1.6–3.1) | 2.8 (2.3–3.6) |
| Liver | 2 | 6 | 26 | 0.3 (0.1–1.1) | 1.4 (0.6–3.4) | 0.7 (0.3–1.5) |
| Gall bladder | 1 | – | 3 | 0.9 (0.1–9.2) | – | 0.6 (0.1–5.5) |
| Pancreas | 18 | 14 | 56 | 1.1 (0.6–1.9) | 1.5 (0.8–2.8) | 1.2 (0.8–1.9) |
| Larynx | 17 | 11 | 48 | 1.3 (0.7–2.3) | 1.4 (0.7–2.7) | 1.3 (0.8–2.2) |
| Lung | 86 | 48 | 252 | 1.2 (0.9–1.6) | 1.1 (0.8–1.6) | 1.2 (0.9–1.4) |
| Bone | 12 | – | 24 | 2.1 (1.0–4.4) | – | 1.3 (0.6–2.7) |
| Melanoma | 4 | 4 | 9 | 1.6 (0.5–5.4) | 2.5 (0.7–8.4) | 2.1 (0.8–5.5) |
| Breast | 58 | 33 | 184 | 1.0 (0.8–1.4) | 1.1 (0.7–1.6) | 1.0 (0.8–1.3) |
| Uterus | 30 | 22 | 89 | 1.1 (0.7–1.7) | 1.5 (0.9–2.4) | 1.2 (0.9–1.7) |
| Ovary | 7 | 6 | 17 | 1.5 (0.6–3.6) | 2.1 (0.8–5.5) | 1.7 (0.8–3.6) |
| Prostate | 17 | 13 | 53 | 1.1 (0.6–1.9) | 1.5 (0.8–2.9) | 1.3 (0.8–2.0) |
| Bladder | 13 | 10 | 42 | 1.0 (0.5–1.9) | 1.5 (0.7–3.1) | 1.2 (0.7–2.0) |
| Kidney | 14 | 3 | 24 | 2.3 (1.2–4.6) | 0.9 (0.3–3.1) | 1.8 (1.0–3.4) |
| Brain | 24 | 10 | 65 | 1.2 (0.8–2.0) | 0.9 (0.4–1.7) | 1.1 (0.7–1.7) |
| Thyroid | 4 | 2 | 10 | 2.2 (0.7–5.5) | 1.5 (0.3–7.4) | 1.7 (0.6–4.7) |
| Lymphomas | 4 | 9 | 22 | 0.7 (0.2–1.9) | 2.8 (1.3–6.3) | 1.4 (0.7–2.8) |
| Leukaemia | 20 | 9 | 59 | 1.2 (0.7–2.0) | 0.9 (0.4–1.8) | 1.1 (0.7–1.7) |
| All sites, excluding intestine | 433 | 258 | 1268 | 1.3 (1.1–1.4) | 1.3 (1.1–1.6) | 1.3 (1.1–1.4) |

[a]Estimates from multiple logistic regression models including terms for age, sex, centre, education and number of siblings (or sisters or brothers). Reference category are the subjects with no history of each particular cancer in first-degree relatives.

**Table 3** Odds ratio[a] (OR) and 95% confidence interval (CI) of colorectal cancer according to history of colorectal cancer in first-degree relatives in strata of selected covariates

| Strata | OR (95% CI) | | |
|---|---|---|---|
| | Colon cancer | Rectal cancer | Colorectal cancer |
| Sex | | | |
| Male | 3.6 (2.5–5.1) | 2.0 (1.2–3.2) | 2.9 (2.1–4.1) |
| Female | 2.9 (2.0–4.2) | 2.5 (1.6–4.0) | 2.9 (2.1–4.0) |
| Age of the proband (years) | | | |
| < 45 | 5.2 (2.1–13.) | 6.3 (1.8–22.) | 5.3 (2.3–12.) |
| 45–59 | 3.3 (2.2–5.0) | 2.2 (1.2–3.8) | 2.9 (2.0–4.2) |
| ≥ 60 | 2.9 (2.1–4.1) | 2.0 (1.3–3.2) | 2.6 (1.9–3.5) |
| Centre | | | |
| Pordenone and Gorizia | 3.3 (2.1–5.1) | 3.5 (2.1–5.9) | 3.4 (2.3–5.1) |
| Milan | 3.0 (1.7–5.1) | 1.4 (0.7–2.8) | 2.3 (1.4–3.7) |
| Genoa | 3.4 (1.7–6.8) | 2.0 (0.7–5.7) | 2.9 (1.5–5.7) |
| Forlì | 1.7 (0.6–4.7) | 1.8 (0.5–6.8) | 1.8 (0.8–4.4) |
| Latina | 4.5 (1.6–12.) | 2.8 (0.8–11.) | 3.8 (1.5–9.5) |
| Naples | 4.4 (2.2–8.8) | 1.2 (0.4–3.9) | 3.3 (1.7–6.3) |
| Years of education | | | |
| < 7 | 3.4 (2.4–4.9) | 2.3 (1.4–3.6) | 3.0 (2.2–4.1) |
| ≥ 7 | 3.0 (2.1–4.3) | 2.3 (1.4–3.7) | 2.8 (2.0–3.9) |

[a]Estimates from multiple logistic regression models including terms for sex, age, centre, education and number of siblings. Reference category is no history of colorectal cancer in first-degree relatives.

cancer in subjects with a family history of any cancer, excluding intestine.

Table 3 presents the ORs of CRC according to family history of intestinal cancer in first-degree relatives in strata of sex, age, centre and education. There was a strong interaction with age of the proband, the OR of colon cancer being 5.2 below age 45 years, 3.3 between 45 and 59 years and 2.9 above age 60 years. For rectal cancer, the corresponding figures were 6.3, 2.2 and 2.0. No clear differences in risk emerged between strata of other covariates.

Various aspects of history of intestinal cancer in first-degree relatives are considered in Table 4. For colon, but not rectal cancer, the presence of two or more affected relatives implied a substantial increase in risk (OR 6.2) in comparison to only one affected relative (OR 3.0). No clear difference in the OR of colon or rectal cancer emerged when the age of diagnosis in the youngest relative was below or over 55 years of age, the OR being 2.9 or 3.3, respectively, for colon and 2.1 in both strata for rectum. When the affected relative was a parent of the proband, the OR was 3.2 for colon, 2.2 for rectum and 3.0 for the two sites together, while the corresponding ORs for only siblings affected were 3.0, 2.2 and 2.7. The OR became 5.4 for colon, 2.0 for rectum and 4.0 for the two sites combined when both parent(s) and sibling(s) were affected.

Table 5 sets out the risks associated with a family history of CRC for different subsites of the tumour in the proband. The OR for CRC family history were 3.4 for the right colon, 3.7 for the transverse and descending colon, 2.5 for the sigmoid colon, 2.9 for the rectosigmoid junction and 2.0 for the rectum; for those with two or more first-degree relatives affected, the ORs were 6.9, 5.8, 3.8, 3.2 and 1.9 respectively.

### DISCUSSION

This study confirms that a family history of intestinal cancer in first-degree relatives increases the risk of both colon and rectal cancer by about threefold, the association being stronger at

© Cancer Research Campaign 1998

**Table 4** Number of cases and odds ratio[a] (OR) of colon and rectal cancer and corresponding 95% confidence interval (CI) according to various aspects of history of colorectal cancer in first-degree relatives (Italy, 1993–96)

| | Number of subjects | | | OR (95% CI) | | |
|---|---|---|---|---|---|---|
| | Colon (*n* = 1232) | Rectum (*n* = 721) | Controls (*n* = 4154) | Colon cancer | Rectal cancer | Colorectal cancer |
| No family history | 1091 | 675 | 4008 | 1[b] | 1[b] | 1[b] |
| Number of affected relatives | | | | | | |
| 1 | 121 | 50 | 139 | 3.0 (2.3–3.9) | 2.2 (1.6–3.1) | 2.7 (2.2–3.5) |
| ≥ 2 | 13 | 3 | 7 | 6.2 (2.4–16) | 2.2 (0.5–8.5) | 4.7 (1.9–11) |
| Youngest age at diagnosis in relatives (years) | | | | | | |
| < 55 | 27 | 11 | 35 | 2.9 (1.7–4.9) | 2.1 (1.0–4.2) | 2.6 (1.6–4.1) |
| ≥ 55 | 100 | 38 | 106 | 3.3 (2.4–4.3) | 2.1 (1.5–3.2) | 2.8 (2.2–3.7) |
| Relative(s) affected | | | | | | |
| Parent(s) | 83 | 30 | 93 | 3.2 (2.4–4.4) | 2.2 (1.4–3.4) | 3.0 (2.2–3.9) |
| Sibling(s) | 46 | 22 | 50 | 3.0 (2.0–4.6) | 2.2 (1.3–3.7) | 2.7 (1.8–3.9) |
| Parent(s) and sibling(s) | 5 | 1 | 3 | 5.4 (1.2–24) | 2.0 (0.2–20.) | 4.0 (1.0–17) |

[a]Estimates from multiple logistic regression models including terms for age, sex, centre, education and number of siblings. [b]Reference category.

**Table 5** Odds ratio[a] (OR) and 95% confidence interval (CI) of colorectal cancer subsites[b] according to history of colorectal cancer in first-degree relatives (Italy, 1993–96)

| | Right colon | | Transverse and descending colon | | Sigmoid colon | | Rectosigmoid junction | | Rectum | |
|---|---|---|---|---|---|---|---|---|---|---|
| | *n* | OR (95% CI) | *n* | OR (95% CI) | *n* | OR (95% CI) | *n* | OR (95% CI) | *n* | OR (95% CI) |
| History of colorectal cancer in first-degree relatives | | | | | | | | | | |
| No[c] | 163 | 1 | 165 | 1 | 402 | 1 | 143 | 1 | 532 | 1 |
| Yes | 22 | 3.4 (2.1–5.4) | 23 | 3.7 (2.3–6.0) | 40 | 2.5 (1.7–3.6) | 16 | 2.9 (1.7–5.1) | 37 | 2.0 (1.3–2.9) |
| Number of affected relatives | | | | | | | | | | |
| 1 | 19 | 3.1 (1.9–5.2) | 21 | 3.6 (2.2–5.9) | 37 | 2.4 (1.6–3.6) | 15 | 2.9 (1.7–5.2) | 35 | 2.0 (1.3–2.9) |
| ≥ 2 | 3 | 6.9 (1.7–28) | 2 | 5.8 (1.2–29) | 3 | 3.8 (0.9–15) | 1 | 3.2 (0.4–27) | 2 | 1.9 (0.4–9.6) |

[a]Estimates from multiple logistic regression models including terms for sex, age, centre, education and number of siblings. [b]The right colon included the hepatic flexure (ICD9 153.0), the caecum (ICD9 153.4), the appendix (ICD9 153.5) and the ascending colon (ICD9 153.6); the transverse (ICD9 153.1) and descending colon (ICD9 153.2) included the splenic flexure (ICD9 153.7); and the other subsites were the sigma (ICD9 153.3), the rectosigmoid junction (154.0) and the rectum (154.1). [c]Reference category.

younger ages and in the right, transverse and descending colon. A few associations emerged with family history of various other cancers, and the OR of CRC was 1.3 for family history of any cancer, excluding CRC.

This study is based on information reported by the subjects, and it is therefore conceivable that cases may recall family history of cancer better than controls. However, recall of cancer history is generally accurate for first-degree, although less for second-degree, relatives (Theis et al, 1994). For this reason, we limited our analysis to first-degree relatives.

Although the use of hospital controls has long been debated (Breslow and Day, 1980), we carefully excluded possible diagnoses for controls potentially related to CRC. Moreover, the hospital setting itself may have improved the comparability of information on medical history (Paganini-Hill and Ross, 1982); participation was practically complete, and the catchment areas of cases and controls were similar. Allowance for major identified potential confounding factors did not materially modify any of the risk estimates.

The major strength and originality of this study, however, is the information on history of cancer at various sites in relatives, and the consequent possibility of obtaining quantitative estimates of CRC risk with reference to family aggregation of other cancers. Additionally, the study was large enough to permit meaningful evaluation of the risk in the subsites of the colon and the rectum.

Our findings are in broad agreement with other reports of an elevated risk of colorectal cancer in subjects with a family history of CRC (Woolf, 1958; Macklin, 1960; Lovett, 1976; Duncan and Kyle, 1982; Bonelli et al, 1988; Ponz de Leon et al, 1989; Sondergaard et al, 1991; La Vecchia et al, 1992; St. John et al, 1993; Fuchs et al, 1994; Goldgar et al, 1994; Slattery and Kerber, 1994; Carstensen et al, 1996; Le Marchand et al, 1996). In most studies, the risk was, as in ours, higher with younger proband age (Lovett, 1976; Sondergaard et al, 1991; La Vecchia et al, 1992; St John et al, 1993; Fuchs et al, 1994; Goldgar et al, 1994; Slattery and Kerber, 1994; Carstensen et al, 1996; Le Marchand et al, 1996). In a joint analysis of the Nurses' Health Study and Health Professionals Follow-up Study cohorts, based on 463 cases of

© Cancer Research Campaign 1998

CRC, the risk conveyed by family history decreased with increasing age of the proband, coming very close to unity above age 65 years (Fuchs et al, 1994). In contrast, in the present study, the risk remained above 2 even over age 65 years, although it was over 5 below age 45 years. A risk above 2 over age 60 years was also observed in a comprehensive population-based study of familial cancer based on the Utah population database (Goldgar et al, 1994). In that study, as in the present one, the risk was also elevated for rectal cancer. No appreciable difference in risk emerged in this study with the age at diagnosis in relatives. Most studies have not reported on the issue, and thus little information is available.

In this study, the associations were similar for subjects with a history of CRC in parents and siblings, thus pointing to an important role of dominant genes (Cannon-Albright et al, 1988; Ponz de Leon et al, 1992).

It has been suggested that the location of the cancer within the colon may determine distinct genetic categories of the disease, in the absence, however, of any quantification of risk (Bufill, 1990). Further, there are differences in the time trends and sex distributions of various subsites (Faivre et al, 1989; Devesa and Chow, 1993). Many studies, however, did not find a higher risk for subjects with a family history in the proximal colon rather than in the distal colon (St. John et al, 1993; Fuchs et al, 1994; Slattery and Kerber, 1994); however the definition of proximal varied as some studies also included transverse colon.

In the present study, when first-degree family history of CRC was considered as a whole, the risk of cancer in the right colon was only moderately higher than that of cancer in the left colon. However, when analysis was limited to individuals with two or more relatives with CRC, the risk approached 7 in the right colon, compared with 4 in the sigmoid colon and 2 in the rectum. These estimates were based on limited numbers and may be due to chance. They however suggest that genes with high penetrance may have a major role in the right colon.

In the Utah population database, moderate but significant increases of colon cancer risk were observed in subjects with a first-degree family history of cancers of the breast, uterus, ovary and prostate (Slattery and Kerber, 1994). Our study found no association with breast cancer, even when analyses were restricted to early age at diagnosis in the proband or in the relative, while in subjects below age 50 years the risks for family history of prostate, uterine and ovarian cancers were 2.5, 1.4 and 18.6 respectively; the last estimate, however, was based only on three cases and one control. One of these three cases had the mother and one sister with ovarian cancer diagnosed at ages 72 and 43 years, respectively, suggesting an involvement of the *BRCA1* gene (Ford et al, 1994).

No strong associations emerged with other digestive or respiratory sites, apart from a borderline significant association of colon cancer with a family history of stomach cancer and an association between lymphomas and rectal cancer. A previous study in northern Italy (La Vecchia et al, 1992) also found an association with gastric cancer, and the Utah Population Database (Goldgar et al, 1994) with leukaemias. The associations with bone and kidney cancer for colon in this study are probably because of misclassification of metastases and/or chance.

We report a 30% higher risk of CRC in patients reporting a history of cancer at any site, excluding intestine, and the risk was similar in both sexes. There may have been some over-reporting of cancer history in families of cases. However, in a companion study on breast cancer using the same design, study areas and questionnaire, the risk of breast cancer associated with family history of cancer at any site excluding breast was close to unity (Negri et al, 1997), and there is no clear reason why CRC cases should tend to over-report cancer in relatives more than breast cancer cases. As in hereditary syndromes involving CRC, such as, for instance, HNPCC, an excess of a variety of other cancers has also been reported, conceivably the weak increase in risk observed may be real. Mortality rates for cancer of the intestine are much lower in the south of Italy than in the north (La Vecchia and Decarli, 1986), and the frequency of HNPCC is also notably lower in the south (Modica et al, 1995). In our study, however, the risk of family history of intestinal cancer was consistent across various geographical areas and, if anything, tended to be higher in the two centres from southern Italy (Latina and Naples), particularly for colon cancer.

In conclusion, this study provides further information on the risk of CRC related to a family history of cancer at several sites and allows a more precise quantification of the risk in different subsites.

### ACKNOWLEDGEMENTS

This work was conducted within the framework of the CNR (Italian National Research Council) applied project 'Clinical Applications of Oncological Research' (contract nos 96.00759.PF39 and 96.00701.PF39), and with the contributions of the Italian Association for Cancer Research and Mrs Angela Marchegiano Borgomainerio. The authors thank Mrs Judy Baggott, Ms M Paola Bonifacino and the GA Pfeiffer Memorial Library staff for editorial assistance.

### REFERENCES

Bonelli L, Martines H, Conio M, Bruzzi P and Aste H (1988) Family history of colorectal cancer as a risk factor for benign and malignant tumours of the large bowel. A case–control study. *Int J Cancer* **41**: 513–517

Breslow N and Day NE (1980) *Statistical methods in cancer research. The analysis of case–control studies.* IARC Sci Publ No 32

Bufill JA (1990) Colorectal cancer: evidence for distinct genetic categories based on proximal or distal tumor location. *Ann Intern Med* **113**: 779–788

Cannon-Albright LA, Skolnick MH, Bishop DT, Lee RG and Burt RW (1988) Common inheritance of susceptibility to colonic adenomatous polyps and associated colorectal cancers. *N Engl J Med* **319**: 533–537

Carstensen B, Soll-Johanning H, Villadsen E, Sondergaard JO and Lynge E (1996) Familial aggregation of colorectal cancer in the general population. *Int J Cancer* **68**: 428–435

Devesa SS and Chow W-H (1993) Variation in colorectal cancer incidence in the United States by subsite of origin. *Cancer* **71**: 3819–3826

Duncan JL and Kyle J (1982) Family incidence of carcinoma of the colon and rectum in north-east Scotland. *Gut* **23**: 169–171

Faivre J, Bedenne L, Boutron MC, Milan C, Collonges R and Arveux P (1989) Epidemiological evidence for distinguishing subsites of colorectal cancer. *J Epidemiol Commun Hlth* **43**: 356–361

Ford D, Easton DF, Bishop DT, Narod SA, Goldgar DE and Breast Cancer Linkage Consortium (1994) Risks of cancer in BRCA1-mutation carriers. *Lancet* **343**: 692–695

Franceschi S, Favero A, La Vecchia C, Negri E, Conti E, Montella M, Giacosa A, Nanni O and Decarli A (1997) Food groups and risk of colorectal cancer in Italy. *Int J Cancer* **72**: 1–6

Fuchs CS, Giovannucci EL, Colditz GA, Hunter DJ, Speizer FE and Willett WC (1994) A prospective study of family history and the risk of colorectal cancer. *N Engl J Med* **331**: 1669–1674

Goldgar DE, Easton DF, Cannon-Albright LA and Skolnick MH (1994) Systematic population-based assessment of cancer risk in first-degree relatives of cancer probands. *J Natl Cancer Inst* **86**: 1600–1608

Houlston RS and Peto J (1996) Genetics and the common cancers. In *Genetic Predisposition to Cancer.* Eeles RA, Ponder BAJ and Easton DF (eds), pp. 208–226. Chapman & Hall: London

© Cancer Research Campaign 1998

La Vecchia C and Decarli A (1986) Cancer mortality in Italy: temporal trends and geographical distribution. *Eur J Cancer* **22**: 1425–1429

La Vecchia C, Negri E, Franceschi S and Gentile A (1992) Family history and the risk of stomach and colorectal cancer. *Cancer* **70**: 50–55

Le Marchand L, Zhao LP, Quiaoit F, Wilkens LR and Kolonel LN (1996) Family history and risk of colorectal cancer in the multiethnic population in Hawaii. *Am J Epidemiol* **144**: 1122–1128

Lovett E (1976) Family studies in cancer of the colon and rectum. *Br J Surg* **63**: 13–18

Lynch HT, Smyrk T, Watson P, Lanspa SJ, Boman BM, Lynch PM, Lynch JF and Cavalieri J (1991) Hereditary colorectal cancer. *Semin Oncol* **18**: 337–366

Lynch HT, Smyrk T and Lynch JF (1996) Overview of natural history, pathology, molecular genetics and management of HNPCC (Lynch syndrome). *Int J Cancer (Pred Oncol)* **69**: 38–43

Macklin MT (1960) Inheritance of cancer of the stomach and large intestine in man. *J Natl Cancer Inst* **24**: 551–571

Modica S, Roncucci L, Benatti P, Gafà L, Tamassia MG, Dardanoni L and Ponz de Leon M (1995) Familial aggregation of tumors and detection of hereditary non-polyposis colorectal cancer in 3-year experience of 2 population-based colorectal-cancer registries. *Int J Cancer* **62**: 685–690

Negri E, Braga C, La Vecchia C, Franceschi S and Parazzini F (1997) Family history of cancer and risk of breast cancer. *Int J Cancer* **72**: 735–738

Paganini-Hill A and Ross RK (1982) Reliability of recall of drug usage and other health-related information. *Am J Epidemiol* **116**: 114–122

Peto J (1980) Genetic predisposition to cancer. In *Cancer Incidence in Defined Populations*. Banbury Report, 4. pp. 203–213. Cold Spring Harbor Laboratory: Cold Spring Harbor

Ponz de Leon M, Sassatelli R, Sacchetti C, Zanghieri G, Scalmati A and Roncucci L (1989) Familial aggregation of tumors in the three-year experience of a population-based colorectal cancer registry. *Cancer Res* **49**: 4344–4348

Ponz de Leon M, Scapoli C, Zanghieri G, Sassatelli R, Sacchetti C and Barrai I (1992) Genetic transmission of colorectal cancer: exploratory data analysis from a population based registry. *J Med Genet* **29**: 531–538

St John DJB, McDermott FT, Hopper JL, Debney EA, Johnson WR and Hughes ESR (1993) Cancer risk in relatives of patients with common colorectal cancer. *Ann Intern Med* **118**: 785–790

Scott RJ and Muller H (1993) Familial and genetic aspects of colorectal carcinogenesis. *Eur J Cancer* **29A**: 2163–2167

Slattery ML and Kerber RA (1994) Family history of cancer and colon cancer risk: the Utah population database. *J Natl Cancer Inst* **86**: 1618–1626

Sondergaard J, Bulow S and Lynge E (1991) Cancer incidence among parents of patients with colorectal cancer. *Int J Cancer* **7**: 202–206

Theis B, Boyd N, Lockwood G and Tritchler D (1994) Accuracy of family cancer history in breast cancer patients. *Eur J Cancer Prev* **3**: 321–327

Woolf CM (1958) A genetic study of carcinoma of the large intestine. *Am J Hum Genet* **10**: 42–52

World Health Organization (1977) *Manual of the International Statistical Classification of Diseases. Injuries and causes of death*. 9th Revision. WHO: Geneva

© Cancer Research Campaign 1998